# Mitochondria Synergize With P2 Receptors to Regulate Human T Cell Function

**DOI:** 10.3389/fimmu.2020.549889

**Published:** 2020-09-29

**Authors:** Carola Ledderose, Wolfgang G. Junger

**Affiliations:** Department of Surgery, Beth Israel Deaconess Medical Center and Harvard Medical School, Boston, MA, United States

**Keywords:** P2X4, mitochondria, inflammation, P2X1, P2Y11

## Abstract

Intracellular ATP is the universal energy carrier that fuels many cellular processes. However, immune cells can also release a portion of their ATP into the extracellular space. There, ATP activates purinergic receptors that mediate autocrine and paracrine signaling events needed for the initiation, modulation, and termination of cell functions. Mitochondria contribute to these processes by producing ATP that is released. Here, we summarize the synergistic interplay between mitochondria and purinergic signaling that regulates T cell functions. Specifically, we discuss how mitochondria interact with P2X1, P2X4, and P2Y11 receptors to regulate T cell metabolism, cell migration, and antigen recognition. These mitochondrial and purinergic signaling mechanisms are indispensable for host immune defense. However, they also represent an Achilles heel that can render the host susceptible to infections and inflammatory disorders. Hypoxia and mitochondrial dysfunction deflate the purinergic signaling mechanisms that regulate T cells, while inflammation and tissue damage generate excessive systemic ATP levels that distort autocrine purinergic signaling and impair T cell function. An improved understanding of the metabolic and purinergic signaling mechanisms that regulate T cells may lead to novel strategies for the diagnosis and treatment of infectious and inflammatory diseases.

## Introduction

ATP is the main energy carrier of living cells. Therefore, it came as a surprise to many when Geoffrey Burnstock first reported that neurons release a portion of their cellular ATP and that the released ATP acts as a signaling molecule for cell-to-cell communication (1). Subsequently, similar ATP signaling mechanisms were identified in many other tissues and organ systems (2, 3). Purinergic signaling enables single cells in a multicellular system to calibrate their individual responses in order to serve the collective interest of the entire organism. Purinergic signaling comprises three basic elements: (i) mechanisms that produce and release ATP into the pericellular space; (ii) purinergic receptors that recognize released ATP and its metabolites and elicit intracellular signals that regulate cell functions; (iii) mechanisms that terminate purinergic signaling by enzymatic breakdown of ATP, cellular re-uptake, or simple diffusion of ATP and its metabolites away from cells.

Intact cells can release ATP via vesicular exocytosis or ATP-permeable membrane channels that include connexin hemichannels, pannexin channels, calcium homeostasis modulator 1, maxi-anion channels, and volume-regulated anion channels (4, 5). Of these mechanisms, pannexin 1 (panx1) channels are particularly important in immune cells (6–10). Under basal conditions, resting cells release only a small portion of their cellular ATP. However, mechanical stimuli or the ligation of cell surface receptors such as the antigen and chemokine receptors of T cells rapidly increase cellular ATP release (10–12). While regulated ATP release fine-tunes cell responses, excessive ATP leakage from dying cells or damaged tissues can act as a danger signal that exacerbates inflammation, impairs T cell functions, and disrupts immune responses (13–16).

ATP release and its breakdown products defines immune cell functions by autocrine stimulation of three different families of purinergic receptors, namely P1, P2X, and P2Y receptors. Different combinations of these receptors are present on the surfaces of virtually all mammalian cells, including the different immune cell subtypes (17). P1 receptors, which recognize adenosine, comprise four subtypes: A1, A2a, A2b, and A3 receptors. P2X receptors recognize ATP and consist of seven members (P2X1-7). Human P2Y receptors comprise eight members that recognize a wider range of ligands (18–21). P2Y2, P2Y4, P2Y11, and P2Y13 receptors are activated by ATP; but certain P2Y receptors also recognize other nucleotides including ADP (P2Y1, P2Y12, P2Y13), UTP (P2Y2, P2Y4, P2Y6), UDP (P2Y4, P2Y6), and UDP-glucose (P2Y14) (21, 22). P1 and P2Y receptors belong to the G protein-coupled receptor (GPCR) superfamily, while P2X receptors are ATP-gated cation channels that facilitate the influx of extracellular Ca^2+^.

Purinergic receptors differ greatly in their desensitization kinetics and affinities for their individual ligands. The extracellular concentrations of these ligands depend on the activities of ectoenzymes expressed on the cell surface (23). Several different groups of these enzymes have been identified including ectonucleoside triphosphate diphosphohydrolases (ENTPDases), ectonucleotide pyrophosphatases/phosphodiesterases (ENPPs), ecto-5′-nucleotidase (CD73), adenosine deaminase (ADA), as well as alkaline phosphatases (23–25). These enzymes are widely distributed among the different immune cell subpopulations (24). CD39 (ENTPD1) that converts extracellular ATP and ADP into AMP, and CD73 that degrades AMP to adenosine are particularly important modulators of purinergic signaling in immune cells (26, 27). Once released from cells, ATP and its breakdown products can either diffuse away from cells or be internalized by equilibrative and concentrative nucleotide transporters that are embedded in the cell membrane and return ATP and its breakdown products for recycling and reuse in cell metabolism (28). The distribution patterns of ATP release sites, ectonucleotidases, and nucleoside transporters along with their relative proximity to P1 and P2 receptors are important determinants of the purinergic signaling mechanisms that regulate immune cell functions.

## P2X1 Receptors Maintain Mitochondrial Metabolism of Quiescent T Cells

**Figure 1:**
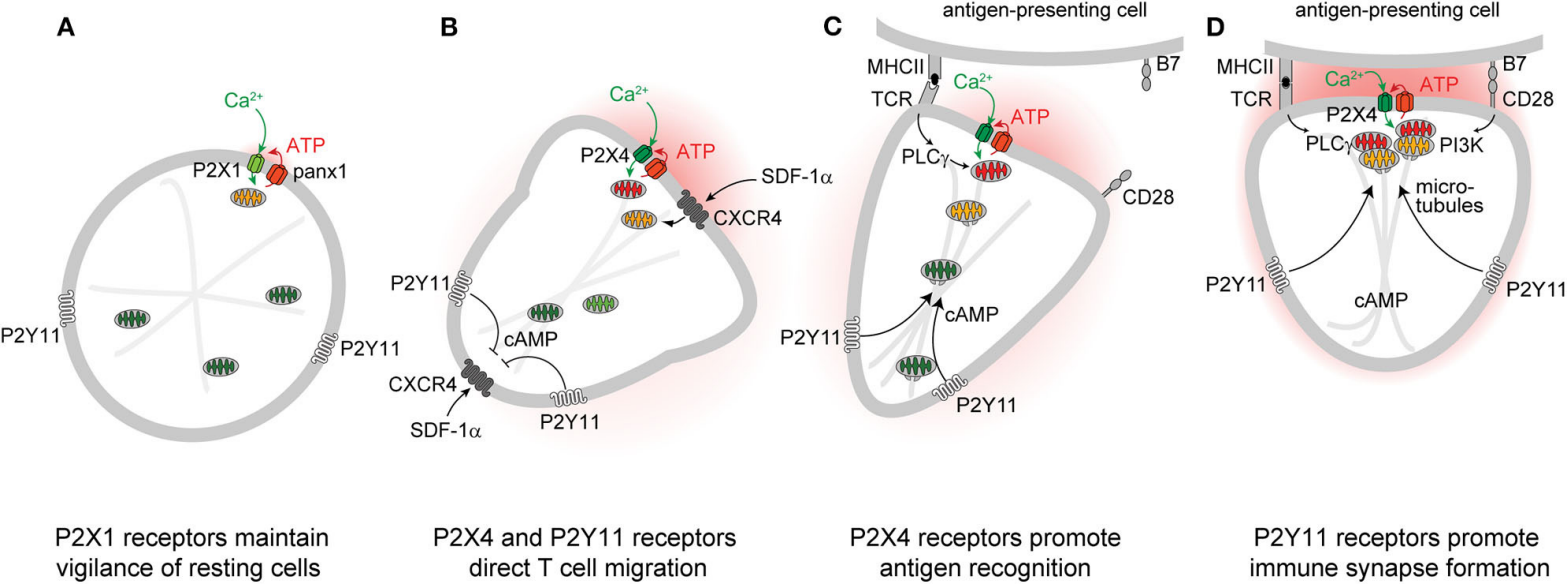
P2 receptors and mitochondria regulate key T cell functions. Autocrine feedback through P2X1 receptors and low-level mitochondrial metabolism maintain a state of vigilance that quiescent T cells need for immune surveillance **(A)**. Chemokine receptors such as CXCR4 trigger mitochondrial metabolism that stimulates P2X4 and P2Y11 receptor-mediated excitatory and inhibitory Ca^2+^ and cAMP signaling pathways that direct cell movement at the front and back of migrating T cells **(B)**. P2X4 receptor accumulation at the immune synapse enhances T cell receptor (TCR) signaling and promotes antigen recognition and the engagement of T cells with antigen-presenting cells **(C)**. P2Y11 receptor recruitment to the uropod of polarized cells induces cAMP/PKA signaling that helps direct the trafficking of mitochondria to the immune synapse **(D)**.

Autocrine purinergic signaling is an important mechanism of immune cell regulation (17, 29–33). Human T cells express A2a, A2b, A3, P2X1, P2X4, P2X5, and P2X7, as well as all eight P2Y receptor subtypes (34–36). P2X1, P2X4, P2Y11, and P2X7 receptors have particularly important roles in the regulation of CD4 T cells (10–12, 36–40). Among these receptors, P2X1 receptors are most sensitive with an EC_50_ value of 50-1000 nM ATP (22, 41). Such ATP levels are well within the concentration range found in the pericellular environment of quiescent T cells (42). Constitutive ATP release from cells overexpressing P2X receptors is sufficient to sustain the modest Ca^2+^ uptake that preserves basal mitochondrial metabolism and ATP synthesis of resting cells (43). P2X1 receptors maintain mitochondrial metabolism in quiescent human CD4 T cells by facilitating cellular Ca^2+^ influx that sustains basal mitochondrial Ca^2+^ levels (44). Inhibition of mitochondrial metabolism and interruption of the electron transport chain impairs T cell migration, indicating that mitochondrial ATP production fuels the purinergic signaling mechanisms needed for immune surveillance and T cell functions (12, 45). Indeed, mitochondrial defects and T cell suppression are cardinal features of sepsis that correlate with morbidity and clinical outcome (44, 46–49). Taken together, these findings suggest that P2X1 receptor-mediated Ca^2+^ influx, mitochondrial ATP production, basal ATP release, and autocrine feedback through P2X1 receptors represent a purinergic-metabolic signaling loop that maintains cell metabolism of quiescent T cells and allows these cells to mount the responses needed for effective host immune defense following chemokine or antigen stimulation (Figure 1A).

## P2X4 Receptors and Mitochondrial Metabolism Promote T Cell Migration

Stimulation of CXCR4, CCR5, CCR7, and other chemokine receptors leads to the recruitment of T cells to lymphoid organs where cell migration enables them to engage and interact with antigen-presenting cells (APCs) (50–52). Stimulation of CXCR4 by stromal cell-derived factor 1α (SDF-1α) causes rapid surges of mitochondrial ATP synthesis and panx1-mediated ATP release from CD4 T cells (12, 53). The resulting pericellular ATP levels trigger P2X4 receptors with an estimated EC_50_ value ranging between 0.5 and 10 μM (22, 41). Autocrine stimulation of P2X4 receptors promotes waves of Ca^2+^ influx that further upregulate mitochondrial ATP synthesis to the levels needed for active T cell migration (Figure 1B) (12). P2X4 receptors aggregate in raft-like structures that associate with mitochondria primarily at the front of migrating T cells where localized ATP synthesis fuels pseudopod protrusion and forward movement of the cells. These P2X4 receptor-driven mechanisms are particularly critical for T cells that move slowly in order to probe their surroundings for potential antigens (12). Faster moving lymphocytes, however, gather their mitochondria primarily at the uropod where the bulk of ATP may be required to fuel actomyosin motor functions needed for rapid cell migration (45). Inhibition of mitochondrial ATP synthesis, ATP release, or P2X4 receptor signaling impairs the ability of T cells to polarize and to migrate in response to CXCR4 stimulation (12, 45, 53).

Similar mitochondrial/purinergic feedback loops also orchestrate the migration of other immune cell subtypes (54–58). Like T cells, neutrophils depend on excitatory purinergic receptors, panx1 channels, and mitochondria to coordinate different aspects of their migration in chemotactic gradient fields (6, 55). However, neutrophils differ from T cells in that P2Y2 receptors rather than P2X4 receptors amplify the chemotactic signals that direct cell migration at their leading edge (6, 54). Microglia, macrophages, and dendritic cells also depend on autocrine feedback mechanisms and specific purinergic receptors to regulate cell migration (56–58). Recent studies have shown that inhibition of the mitochondrial electron transport chain impairs the motility of neutrophils in zebrafish (59). Thus, mitochondrial metabolism and purinergic signaling seem to be preserved features that regulate immune cell migration in humans and other vertebrates.

## P2Y11 Receptors Contribute to T Cell Migration by Restraining Mitochondrial Metabolism

According to the local excitation—global inhibition (LEGI) model of chemotaxis, excitatory mechanisms at the front elicit cell protrusion, while inhibitory mechanisms at the back promote the retraction of the cell body during cell migration (60–62). In neutrophils, P2Y2 receptors provide the excitatory signal at the front, while A2a adenosine receptors generate the inhibitory cAMP/PKA signal that causes cell retraction at the back of cells (63). In T cells, P2X4 and P2Y11 receptors fulfill similar roles in the regulation of cell migration (12, 64). Like the A2a receptors of neutrophils, the P2Y11 receptors of T cells can couple to Gα_s_ proteins that trigger cAMP/PKA signaling pathways (65). P2Y11 receptors bind their natural ligand, ATP, with a reported EC_50_ value of 2.5 to 63 μM, which is similar to the affinity of P2X4 receptors (41). Therefore, the pericellular ATP that surrounds stimulated T cells can trigger both P2X4 receptor-mediated Ca^2+^ influx and P2Y11 receptor-mediated cAMP/PKA signaling that restrains excitatory signaling and transduction pathways downstream of Gα_i/o_-coupled GPCRs like CXCR4 (66, 67). We found that P2Y11 receptors redistribute to the back of polarized T cells where they induce cAMP/PKA signaling events that stabilize cell polarization by locally restricting cell stimulation by CXCR4 chemokine receptors at the back (Figure 1B) (64). Thus, P2X4 and P2Y11 receptors synergize to regulate mitochondrial metabolism and provide T cells with the local excitation and global inhibition cues that organize pseudopod protrusion and uropod retraction during T cell migration in a LEGI-type fashion.

## P2Y11 and P2X4 Receptors Orchestrate the Accumulation and Activation of Mitochondria at the Immune Synapse of T Cells

T cells must interact with APCs in order to mount immune responses. These interactions occur via organized structures referred to as immune synapses (IS) that consist of microclusters containing T cell receptors (TCR), CD3, CD28 co-receptors, LAT, SLP76, LFA-1, microtubules, and other cytoskeletal components (68). The formation of a stable IS between a T cell and an APC enables sustained TCR signaling that culminates in cytokine production and T cell proliferation (69). Efficient T cell activation also depends on sustained Ca^2+^ influx from the extracellular space (70). Just minutes after TCR stimulation, P2X4 receptors, panx1 channels, and mitochondria accumulate at the IS where mitochondria generate the ATP that panx1 channels release into the synaptic cleft to stimulate P2X4 receptor-mediated Ca^2+^ influx (36, 71, 72). P2X4 receptors deliver the Ca^2+^ that mitochondria need to synthesize ATP via oxidative phosphorylation (73). However, mitochondria also act as Ca^2+^ sinks that fine-tune cytosolic Ca^2+^ levels for efficient T cell activation (74). Thus, mitochondria, panx1, and P2X4 receptors represent a powerful feedforward signaling system that triggers downstream pathways that involve mitogen-activated protein kinases (MAPKs) and nuclear factors of activated T cells (NFAT) and induce IL-2 transcription and T cell proliferation (10, 11, 36).

Successful T cell activation depends on the accumulation of mitochondria at the IS (71, 72, 75). However, the mechanisms that orchestrate mitochondrial trafficking to the IS are not clear (76). In neurons, kinesin and dynein motors accomplish anterograde and retrograde trafficking of mitochondria along microtubules (77). In T cells, dynein facilitates mitochondrial transport to contact sites that T cells form with endothelial cells during their transmigration across blood vessel walls (78). Dynamin-related protein 1 (DRP1) is a mitochondrial fission factor that helps direct mitochondria to the uropod of migrating T cells and to the IS during APC engagement (45, 75). In neurons, cAMP promotes directional movement of mitochondria along the microtubule network (79–82), while local cytosolic Ca^2+^ hotspots act as mitochondrial stop signals (83). Our recent work has shown that P2Y11 receptors promote trafficking of mitochondria to the IS of T cells (84). Thus, P2Y11 and P2X4 receptors jointly recruit and activate mitochondria at the IS in order to sustain T cell activation. However, further studies are needed to reveal the detailed mechanisms by which these purinergic receptors, motor proteins, and the microtubule network regulate the complex process that energizes the IS in T cells (Figures 1C,D).

Several lines of evidence indicate that purinergic signaling has important physiological implications for *in vivo* T cell functions. Consistent with the critical roles of P2X receptors in T cells, genetic variants of P2X4 and P2X7 receptors were found to contribute to multiple sclerosis, a T cell-mediated inflammatory autoimmune disease (85). Furthermore, CD4 T cell infiltration into the spinal cord of mice subjected to experimental autoimmune encephalomyelitis is attenuated in *Panx1* knockout mice (53). The significance of P2Y11 receptors as regulators of human immune responses is supported by recent findings that single nucleotide polymorphisms (SNPs) in the P2Y11 receptor gene are associated with inflammatory disorders that increase the risk of acute myocardial infarction and predispose patients to narcolepsy and reduced T cell viability (86, 87).

## Systemic ATP Accumulation Impairs Immune Cell Functions by Interfering With Their Autocrine Purinergic Signaling Mechanisms

**Figure 2:**
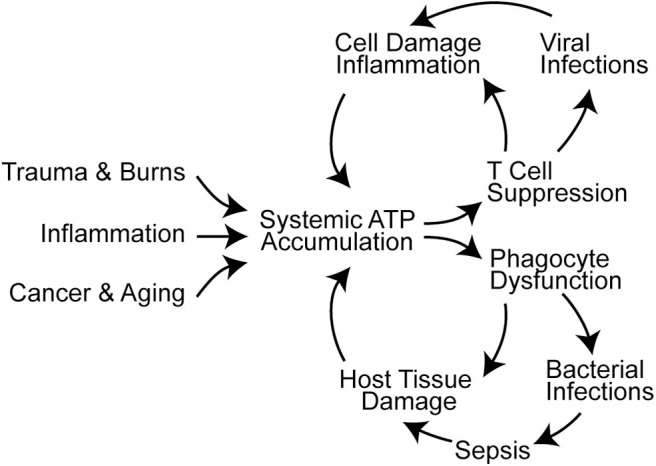
Systemic ATP accumulation impairs the autocrine purinergic signaling mechanisms that regulate immune functions. Trauma, burns, inflammation, cancer, and aging are associated with systemic ATP accumulation that promotes immune cell dysfunction (16, 88–90). This results in infections, sepsis, and additional cell damage that exacerbates systemic ATP levels and propagates immune dysfunction.

T cells travel to lymphoid organs and other host tissues where they interact with APCs in order to elicit effector functions needed for host defense. As outlined above, T cell functions depend on intricate autocrine signaling mechanisms to execute their roles in host defense. However, these autocrine signaling mechanisms are susceptible to paracrine interference by exogenous ATP that accumulates in response to cell damage, tissue injury, or inflammation. Systemic ATP levels also increase in sepsis and in the tumor microenvironment, which impairs T cell migration, cytokine production, and T cell proliferation (Figure 2) (16, 88, 91–93). Global and disproportionate stimulation of P2X1, P2X4, and P2Y11 receptors across the cell surface disrupts the spatiotemporal sequence of the autocrine purinergic signaling events that regulate T cells and host immune functions (64, 94).

Besides P2X1 and P2X4 receptors, T cells also express the P2X7 receptor subtype. P2X7 receptors are comparatively insensitive to ATP with an EC_50_ value of ~780 μM (41). Interestingly, P2X7 receptors remain uniformly distributed across the cell surface of T cells even during IS formation with APCs (36). This suggests that P2X7 receptors may act primarily as mediators of paracrine rather than autocrine ATP signaling. P2X7 receptor stimulation by external ATP can alter the composition of T cell subpopulations by promoting the Th1/Th17 differentiation of CD4 T cells, the conversion of immunosuppressive regulatory T cells (T_regs_) into proinflammatory Th17 cells, and the formation of long-lived CD8 memory T cell subsets (37, 95). However, P2X7 receptors may also contribute to the onset of autoimmune diseases such as type 1 diabetes, namely by enhancing the activation of autoreactive CD8 effector T cells (96). P2X7 receptors differ from other purinergic receptors in that they form large and unselective macropores in response to millimolar ATP concentrations, which ultimately results in cell death (33). Physiologically, this enables P2X7 receptors to control T follicular helper (Tfh) cell numbers in Peyer's patches of the small intestine and to modulate the production of IgA that shapes the gut microbiota composition (97). P2X7 receptor stimulation also limits the expansion of autoreactivity-promoting Tfh cells, whereas Tfh cells that respond to cognate antigens are protected from P2X7 receptor-mediated cell death (97–99). On the other hand, P2X7 receptor-mediated cell death may also contribute to the suppression of T cell immunity in the presence of pathologically elevated systemic ATP levels.

Excessive ATP in the systemic environment of neutrophils has similarly disruptive implications on cell functions. Overstimulation of excitatory P2Y2 receptors disrupts neutrophil chemotaxis and bacterial clearance. At the same time, excessive P2Y2 receptor stimulation by systemic ATP aggravates inflammatory neutrophil responses such as oxidative burst and degranulation, which culminate a in neutrophil-mediated collateral host tissue damage (Figure 2) (100–102). Systemic ATP may have a similar impact on other immune cells including macrophages that depend on P2X4 and P2X7 receptors for bacterial clearance in polymicrobial sepsis (103, 104). Targeting extracellular ATP could be a promising approach to overcome systemic inflammation and immunosuppression in critical care and cancer patients. The therapeutic potential of this approach is supported by observations that treatment with apyrase and other enzymes that hydrolyze extracellular ATP can indeed improve outcome in mouse models of inflammation and sepsis (89, 102, 105).

## Concluding Remarks

Breakdown of increased systemic ATP levels can elevate extracellular adenosine concentrations. Adenosine exerts mostly anti-inflammatory effects through A2a and A2b receptors. While adenosine can protect tissues from inflammatory damage, excessive adenosine signaling contributes to immunosuppression in cancer and sepsis (106). The suppressive effect of A2a receptor stimulation on various T cell functions has been studied in great detail in mice (107). CD39 and CD73 are dominant enzymes responsible for the conversion of ATP to adenosine. Both ectonucleotidases are highly expressed by murine T_regs_ that suppress T cell functions by generating adenosine and stimulating A2a receptors (27, 32). In contrast to mice, CD39 expression on human CD4 T cells is largely restricted to memory T_regs_ (108), and T cell inhibition by adenosine receptor-dependent pathways seems to be less important in humans than in mice (109). Interestingly, mice and other rodents do not possess P2Y11 receptors (110). Thus, mouse models cannot fully reflect human disease processes. It seems likely that A2 adenosine receptors in mice fulfill the roles of human P2Y11 receptors in the regulation of T cell functions. These species-specific differences must be considered during the development of treatments for inflammatory, infectious, and other T cell-centered diseases such as cancer.

## Author Contributions

CL and WJ prepared the manuscript. All authors contributed to the article and approved the submitted version.

## Conflict of Interest

The authors declare that the research was conducted in the absence of any commercial or financial relationships that could be construed as a potential conflict of interest.
